# Monitoring of Caged Bluefin Tuna Reactions to Ship and Offshore Wind Farm Operational Noises

**DOI:** 10.3390/s21216998

**Published:** 2021-10-21

**Authors:** Vicente Puig-Pons, Ester Soliveres, Isabel Pérez-Arjona, Victor Espinosa, Pedro Poveda-Martínez, Jaime Ramis-Soriano, Patricia Ordoñez-Cebrián, Marek Moszyński, Fernando de la Gándara, Manuel Bou-Cabo, José L. Cort, Eladio Santaella

**Affiliations:** 1 Intitut d’Investigació per a la Gestió Integrada de Zones Costaneres, Universitat Politécnica de València, C/Paranimf 1, Grau de Gandia, 46730 València, Spain; essogon@upv.es (E.S.); iparjona@upv.es (I.P.-A.); vespinos@fis.upv.es (V.E.); 2 Unidad Mixta de Investigación en Tecnología para Estudios Marinos IEO-UPV (UTEM), Muelle Frutero, Port de Gandia, Grau de Gandia, 46730 València, Spain; fernando.delagandara@ieo.es (F.d.l.G.); manuel.bou@ieo.es (M.B.-C.); jose.cort@ieo.es (J.L.C.); esantael@gmail.com (E.S.); 3 Institut Universitari de Física Aplicada a les Ciències i les Tecnologies, Universitat d’Alacant, Ap. de Correus 99, 03080 Alacant, Spain; pedro.poveda@ua.es (P.P.-M.); jramis@ua.es (J.R.-S.); 4 Zunibal S.L., Idorsolo Kalea, 1, 48160 Derio, Spain; patricia.ordonez@zunibal.com; 5 Faculty of Electronics, Telecommunications and Informatics, Politechnika Gdańska, Gabriela Narutowicza 11/12, 80233 Gdańsk, Poland; marmo@eti.pg.gda.pl; 6 Instituto Español de Oceanografía, Centro Ocenográfico de Murcia, C/Varadero, 1, San Pedro del Pinatar, 30740 Murcia, Spain

**Keywords:** underwater noise, bluefin tuna, offshore windmill, behavior, anthropogenic impact

## Abstract

Underwater noise has been identified as a relevant pollution affecting marine ecosystems in different ways. Despite the numerous studies performed over the last few decades regarding the adverse effect of underwater noise on marine life, a lack of knowledge and methodological procedures still exists, and results are often tentative or qualitative. A monitoring methodology for the behavioral response of bluefin tuna (*Thunnus thynnus*) when exposed to ship and wind turbine operational noises was implemented and tested in a fixed commercial tuna feeding cage in the Mediterranean sea. Fish behavior was continuously monitored, combining synchronized echosounder and video recording systems. Automatic information extracted from acoustical echograms was used to describe tuna reaction to noise in terms of average depth and vertical dimensions of the school and the indicators of swimming speed and tilt direction. Video recordings allowed us to detect changes in swimming patterns. Different kinds of stimuli were considered during bluefin tuna cage monitoring, such as noise generated by feeding boats, wind farm operational noise, and other synthetic signals projected in the medium using a broadband underwater projector. The monitoring system design was revealed as a successful methodological approach to record and quantify reactions to noise. The obtained results suggested that the observed reactions presented a strong relationship with insonification pressure level and time. Behavioral changes associated with noise are difficult to observe, especially in semi-free conditions; thus, the presented approach offered the opportunity to link anthropogenic activity with possible effects on a given marine species, suggesting the possibility of achieving a more realistic framework to assess the impacts of underwater noise on marine animals.

## 1. Introduction

Human activities cause pressure on the marine environment, affecting it in numerous ways. One of the activities that has increased during the last decades is marine traffic. Some authors have pointed out that from 1992 to 2012, marine traffic has increased at global level of almost 60%. Some areas even experienced an increase ranging from 100% to 200% [1]. Commercial globalization has led to an increase in goods traffic through oceans and seas around the world. Related not only to marine traffic but also other types of human activities, underwater noise has captured a considerable amount of attention as a pollutant, being promoted to the category of threat and widely studied by different organizations, such as ACCOBAMS (Agreement on the Conservation of Cetaceans of the Black Sea, Mediterranean Sea and contiguous Atlantic Area), ASCOBAMS (Agreement on the Conservation of Small Cetaceans of the Baltic, North East Atlantic, Irish and North Seas), IFAW (International Fund for Animal Welfare), and IMO (International Maritime Organization), among others. Many actions have been taken by both Europe and the USA with the aim of establishing standards and methodologies to assess the threat that underwater noise represents [2,3]. One of the more ambitious actions developed during the last years with respect to the study of the marine environment is the Marine Strategy Framework Directive (hereafter referred to as MSFD) adopted in 2008 by EU member states. The main aim of MSFD is to protect the marine ecosystem and biodiversity by establishing the concept of Good Environmental Status (or GES). The directive defines GES as “the environmental status of marine waters where these provide ecologically diverse and dynamic oceans and seas which are clean, healthy and productive”. GES assessment is conducted using eleven qualitative/quantitative descriptors. That related to underwater noise is defined in Descriptor 11 (D11), which accounts for impulsive or continuous noise separately due to the differences among sources and effects produced. The framework related to the assessment of underwater noise typically considers the acoustic sources and sound pressure level generated and propagated through the medium, but, ultimately, the studies linked to the effect of marine biota due to noise are gaining relevance. To perform studies examining the risk of the effect linked to underwater noise, it is necessary to know the influence of sound on animal life. According to [4], most noise effect studies are related to fish, specifically 52%, while 21% correspond to marine mammals. The remaining studies are based on reptiles, mollusks, and arthropods. It is commonly accepted that all fish studied to date are sensitive to noise [5,6,7], and this was confirmed by using two sensory systems depending on the species: the inner ear and the lateral line system. Despite the numerous studies considering different kinds of animals and types of noise, there exists a lack of research with respect to underwater noise effects due to the vastness of the theme. In this work, we report on the behavioral changes observed in bluefin tuna due to continuous noise emitted by an underwater projector. One of the most relevant sources of impulsive noise is related to the installation of turbines in offshore wind farms. The effect of piling noise on fish has been investigated in regard to some target species, with a variety of results, from no evidence of injury or reaction [8,9] to immediate death [10], but fewer studies are related to operational continuous noise. However, different works have addressed the characterization and possible effects of the operational noise of marine turbines on marine life. The effects of installation and operational phases on marine mammals were investigated in [11], cetaceans being the initial focus. Attention was also paid to the effects of such noise on fish and invertebrates, as well as to all of the potential impacts of operating windmills and their emitting characteristics (see, for instance, [12,13,14,15], and references therein). Bluefin tuna (*Thunnus thynnus*) is an emblematic species and also a high value economic resource, and, thus, it has been the subject of many studies over the last decade due to the possible worldwide extinction at the turn of the century. Even assuming the increasing interest in the impact of anthropogenic noise on marine life and the general concern regarding bluefin tuna, only a few studies have attempted to characterize the hearing threshold of similar species such as *Thunnus orientalis* [16], and, thus, there exists a lack of knowledge regarding bluefin tuna as a receptor of noise pollution. Bluefin tuna form schools that migrate at ocean scales crossing the Gibraltar strait from Atlantic Ocean to Mediterranean sea. Their migration routes pass nearby coasts, these regions representing the main candidates to host offshore facilities such as wind farms. The aim of the present work is to contribute to the knowledge of the potential effects of the operational noise of wind turbines on the behavior of bluefin tuna, which could affect their feeding and reproductive migration. Additionally, as a first step, we aim to validate methodologies with semi-captive tuna to study such effects. In order to investigate reactions to operational turbine noise, bluefin tuna located in a feeding cage off the Mediterranean coast were exposed to wind turbine noise and other recordings. The animals were previously monitored for a number of weeks using active acoustics and video systems to ensure that the possible reactions after noise emission were distinguishable from the usual behavior of the fish. Behavioral experiments in cages cannot be directly extrapolated to wild bluefin tuna, and similar methodologies used with other pelagic species [17] or with species with more limited geographical movements should be applied [18,19]. An example of this is the use of acoustical and other types of sensing tagging (dive loggers) that have been successfully applied to harbor porpoises to investigate the relationships between their reaction and noise sources. However, monitoring tuna is extremely difficult because of their great mobility and the required delicate manipulation, mainly in wild conditions but also in the semi-captive condition presented here [20]. Our experiment was deemed an exceptional opportunity due to the potential implication for tuna farming. The presence or absence of a response of animals to particular stimuli, in this case, an acoustical disturbance, depends on different factors, such as stress, individual characteristics, previous experience, and the presence of prey or predators, among others. The obtained results on semi-captive tuna must be interpreted carefully, but the observed effects caused by wind turbine noise could allow us to interpret future reactions observed in the wild.

## 2. Materials and Methods

### 2.1. Location and Measurement Conditions

Activities reported via this communication were carried out at facilities located in L’Ametlla de Mar (latitude = 40°52′11.7″ N and longitude 0°48′15.2″ E), Mediterranean Sea. The experiment was performed from 23 to 25 July 2013. Bluefin tuna are usually caught along their migration route, close to the Balearic Islands in the Mediterranean Sea. Animals after capturing by purse seiners were transferred to floating cages and towed during a period of 10–15 days from the Balearic coastal region to the fattening farms, located near the continental coast traveling about 200 km at a speed of 1 knot. The dimensions of the final feeding cage were 50 m in diameter and 28 m in depth. The total number of fish was approximately 900 bluefin tuna, weighing 200 kg on average and caught during the first week of June 2013. During their stay in the cages until the time of the experiment, the tuna were subjected to ship operational acoustical stimuli, which must be taken into account when discussing the results of the work; the presence of feeding boats twice per day and other operational works (cleaning, repairing, surveillance, etc.) will most likely alter the response of semi-captive tuna, thereby distinguishing them from the wild ones. Moreover, during the trip from Balearic waters, the tuna were continuously subjected to the towing boat noise, an aspect that should be also taken into account.

### 2.2. Background Tuna Behavior

**Figure 1:**
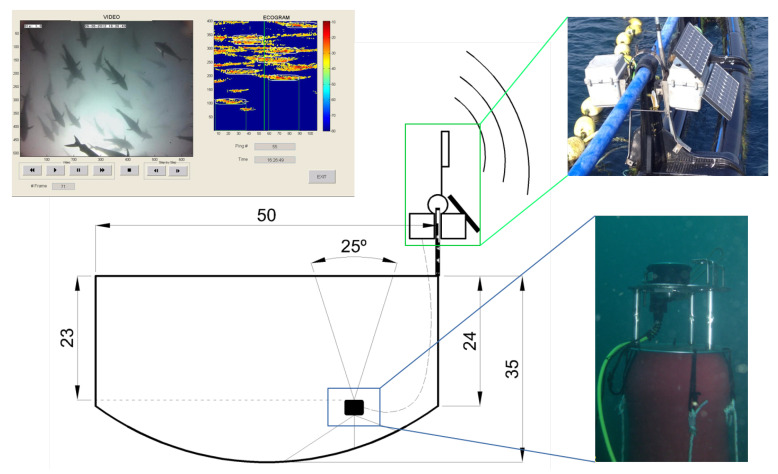
Scheme of the continuous long-term monitoring system, composed of a control echosounder, a video camera, and a real-time transmission system. A floating platform with the video camera and the ultrasonic transducer was placed at a depth of 24 m and cabled to the surface where the autonomous echosounder and communication systems were placed in a waterproof box. The solar power electronics and batteries were placed in a second box together in a structure fixed to the cage rim. The resulting synchronized video and echogram are also depicted.

In order to study tuna behavior in sea cages in normal production conditions, the behavioral patterns of the tuna school in the farm were continuously monitored for 6 weeks from January to February 2013. The studied tuna were caught during the fishing season in June 2012. The acoustical recording system consisted of an autonomous single-beam Knudsen Engineering ROVER echosounder (Figure 1), working at 200 kHz and covering an angle of 25° at −3 dB from the maximum emission level on the transducer axis. The transducer was located facing upwards toward the surface at a depth of 24 m at the bottom of a floating commercial cage that was 50 m in diameter and anchored to the middle of the trap radius. In addition, an underwater video camera system was installed, working together with the acoustic system and creating a redundant system of monitoring. The system was powered by external batteries and solar panels located on the cage and installed inside a waterproof box. Data transfer was ensured by a Wi-Fi link to the shore providing the possibility of remote control of the system and real-time acquisition. Nevertheless, all data were also stored locally on the computer incorporated by the Knudsen echosounder. This combination of video cameras and echosounders for the behavioral monitoring of fish when exposed to underwater noise was also used by [21] in herring cages. Later, a combination of two different echo sounding systems (vertical echosounder and sidescan sonar) was also used by [17] for behavioral response observations in the wild.

### 2.3. Exposure to Pure Tones, Synthetic Noises, and Hydrophone Recording Playback

**Figure 2:**
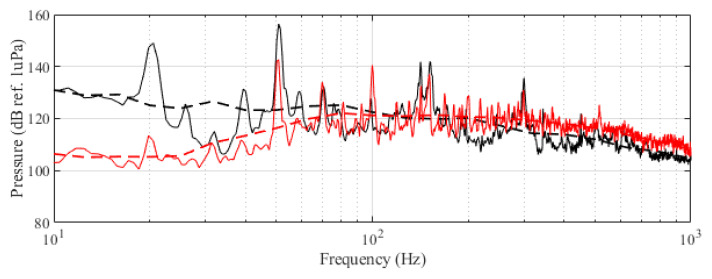
Original recorded turbine emission (black) and reproduced noise used in the experiment (red). Dashed curves correspond to the third-octave levels (dB ref 1 μPa RMS) of the same signals.

**Figure 3:**
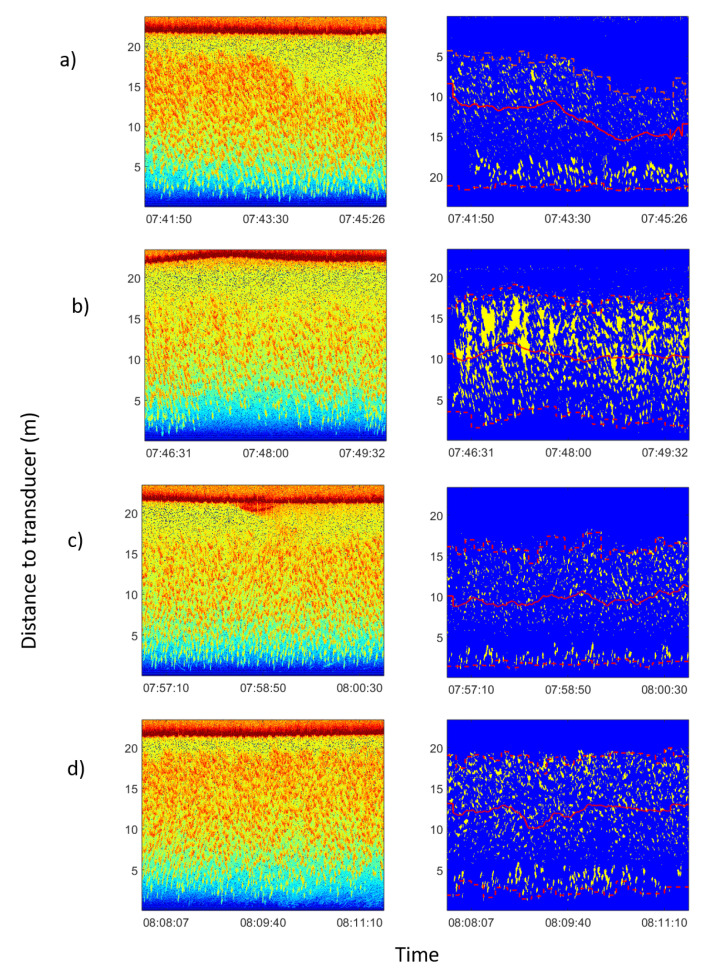
Echograms corresponding to feeding process on 15 January 2013. In the left column raw echograms are shown. In the rigth column echograms of the school had been isolated from noise following the procedure explained in Section 2.4. Average school depth and upper and lower limits were calculated automatically. In all figures the red solid line represents the average depth of the school; the lower dashed line represents the lower limit of the school; and the upper dashed line represents the upper limit of the school. From the inspection of the processed echograms the change in the vertical distribution of fish can be observed as a consequence of boat operations: (**a**) boat approached, and school dived at an average distance from transducer of 12.2 m (before 07:44:00) to 8.9 m (after 07:44:00). (**b**) Boat moored and unloaded food block. Tuna dived deeper to 10.4 m. (**c**) Food block through the acoustic beam. School dived deeper to 9.7 m. (**d**) Boat departed from the cage and tuna rose again at an average distance from the transducer at the bottom of 12.3 m.

**Figure 4:**
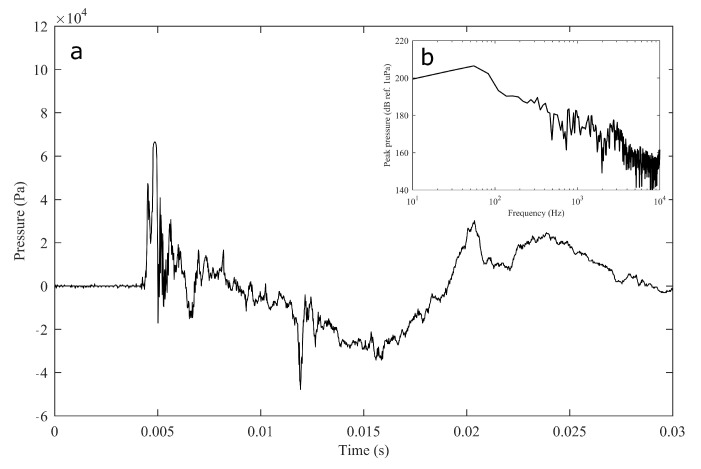
Impulsive noise temporal recording of a lupara used for tuna slaughter (**a**) and its spectral content (**b**, inset).

The experiment was originally designed to test the effect of operational turbine noise on bluefin tuna behavior, but other sounds were also reproduced for a better understanding of the possible tuna reactions and validation of the observing system. The noise of a wind turbine was previously recorded at 50 m from the source for 30 s and sampled at 350 kHz. It is possible to observe the spectrum of the wind turbine in Figure 2. The sound could be understood as a broad band noise with similar levels, around 120 dB ref 1 μPa, along the whole spectrum defined within 30 Hz–10k Hz, with a maximum sound pressure level centered at 50 Hz (142 dB ref 1 μPa). Considering the audibility threshold of similar species and the sound pressure level generated by the source, bluefin tuna were expected to react to the projected sound, even more so when maximum levels overlapped with the frequency range of sound generated by tuna, probably produced by swimbladder contraction [22]. The emitter used to project the recorded noise to the medium was Data Physics GW350, an underwater sound projector provided by the Spanish Navy. This device is specially designed to simulate the acoustic signatures of specific sonar targets. Typically, it is used to train the crews of surface ships, submarines, and helicopters in anti-submarine warfare techniques. The source allowed the emission of low-frequency sound in the band of 20 Hz to 3200 Hz, suitable for the pursued objectives. The projector was assembled in a metal structure, implementing a pressure compensator in order to increase the operation depth of the source. The system was deployed to a depth of 10 m by means of a crane. The projector was connected to a 300 W power amplifier into which a signal was fed from an National Instruments PXI-5412 100 MS/s signal generator connected to a laptop computer. The electric power was provided by the boat battery array, and during the measurements, both the main ship’s engine and the auxiliary electric generator were switched off. Sound pressure level measurements during the signal’s playback were made by means of two calibrated hydrophones, an ITC1032 (sensitivity: −194 dB ref 1 V/μPa, ranging from 10 Hz to 50 kHz) and a B&K 8103 (sensitivity: −211 dB ref 1 V/μPa, from 0.1 Hz to 180 kHz), located at distances of 25 m and 10 m from the source, respectively. The recorded signals were amplified using a B&K Nexus Signal Conditioner Type 2693 and digitized using an NI PXI-5102 oscilloscope at sample rates of 44,100 Hz and 96,000 Hz, and data were stored on a laptop computer. The sound pressure level (SPL) was calculated for each frequency applying an exponential RMS averaging (expressed as dB ref 1 V/μPa). The emitted signals were classified in three different groups: single tones, synthetic broadband noises, and recorded noises. The first group was composed of pure tones of 30 Hz, 50 Hz, 100 Hz, 150 Hz, 200 Hz, 300 Hz, 500 Hz, 1000 Hz, and 4000 Hz. The amplitude of each tone could be as high as 137, 150, 162, 160, 155, 158, 152, 155, or 150 dB (ref 1 V/μPa), respectively. The second group, synthetic broadband noises, consisted of four different signals: white noise (average SPL 120 dB), maximum length sequences (MLS) (125 dB), time-stretched pulses (TSP) (150 dB), and sine sweeps (140 dB) [23]. The last group consisted of recorded submarine noises, produced by tuna farm ships (Figure 3) and the previously mentioned recording of an offshore wind turbine in operation (Figure 2). Additionally to continuous noise, a recording of an impulsive noise, regular in the farm acoustic landscape, was registered. This last recorded sound came from a lupara shot, which is a tool commonly used in the slaughter of tuna with a peak SPL above 216 dB ref 1 μPa (Figure 4). Previously to our experiment, it was visually observed that fish reacted with sudden accelerations when an individual slaughter was made.

**Figure 5:**
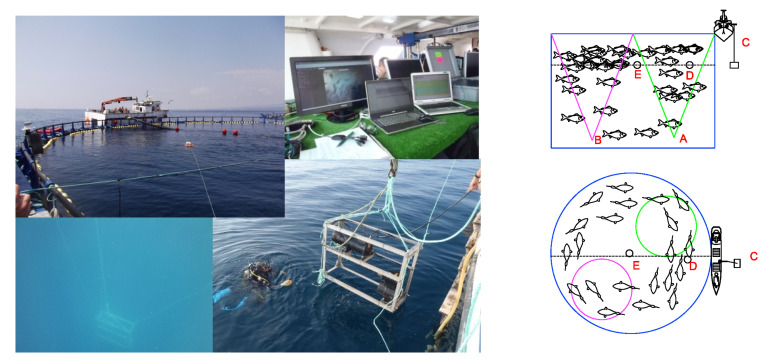
**Right panel**: Setup used for insonification experiments. The Knudsen echosounder and the video camera were placed at point A in the diagram. The Biosonics echosounder was placed at point B, also looking up toward the sea surface. The underwater projector was suspended from the observation boat at point C, and two calibrated hydrophones were positioned at points E and D. **Left panel**: Image of operations with underwater projector being installed and the control desk with both image and acoustic real-time observations.

During the exposure to the turbine noise, an extra single-beam DT-X Biosonics scientific echosounder (Figure 5), also working at 200 kHz and an aperture at −3 dB of 20°, was installed at the opposite side of the cage to gather more information about the school’s distribution and behavior. The video camera was located alongside the Knudsen echosounder (Figure 5).

### 2.4. Data Analysis

Acoustic data were analyzed automatically using software developed specifically for this purpose in the framework of Matlab(R). First, data were split using two criteria according to the intended end use. In the case of background tuna behavior, data were divided into 1-hour files for subsequent analysis. In the noise exposure case, data were divided into events. Every event refers to one sound stimulus to which tuna were subjected (pure tones and synthetic and recorded noises).

The behavior was characterized in terms of three variables:

- Average depth and upper and lower limits of the school;
- Average length of traces;
- Average tilt of traces.

**Figure 6:**
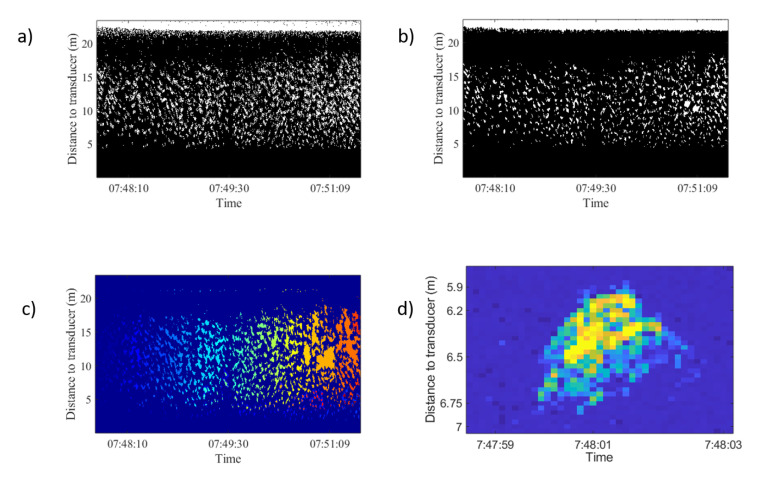
Image processing thechniques used and tested in Figure 3 top-right echogram. (**a**) Image binarization. (**b**) Morphological operation applied: thickening to provide more compact regions, opening to remove protrusions (noise), breaking weak connections, and closing to smooth out contours and fill small holes. (**c**) Region-based segmentation results. (**d**) Isolated trace from this school.

**Figure 7:**
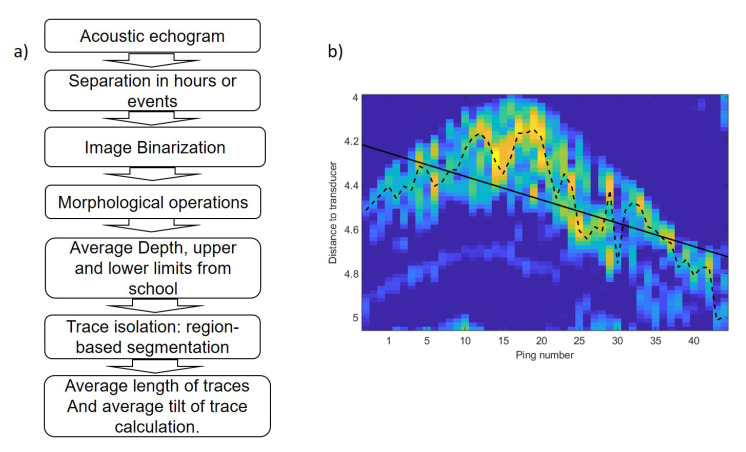
(**a**) Sequence of data processing algorithm. (**b**) Trace of fish swimming upward. Dotted line shows the distance of maximum backscattering value for each ping. The solid line represents a linear fit applied to range values shown by the dashed line.

The average depth of the school was calculated similar to the center of mass of the acoustic volume backscattering strength following the method of [24] and references therein. To analyze trace length and tilt, digital imaging processing techniques were used to transform the echogram into a binary image using the threshold level method [25]. In order to obtain isolated traces, a sequence of morphological operations was applied to achieve more compact regions and to remove noise [26]. Figure 6 shows the image binarization, morphological operations, region-based segmentation and trace isolation of school presented in Figure 3. In regard to this, to obtain data from the school, the average depth and upper and lower limits were calculated for every ping. Based on the ping average data, an average value per hour was calculated in the behavior monitoring experiment and an average value of three parameters was estimated for each event. Afterward, region-based segmentation was carried out. To separate tuna traces from unwanted targets, a region size threshold and an echo level threshold were used. The geometrical parameters of each trace were stored with its distance to the transducer and its duration. Acoustic data processing produced a collection of traces for each hour or event. Finally, the tilt of traces was calculated using the maximum backscattering value of each ping of the trace and the distance from each maximum to the transducer (Figure 6b). A linear fit was applied to the range values and the slope of the line was used as a tilt of the trace indicator. The tilt of traces was used to assess whether tuna swam upward or downward. The sequence of acoustic data processing algorithms is shown in Figure 7a.

### 2.5. Statistics Analysis

Statistical analysis was carried out to analyze the suitability of measured variables to describe the tuna’s reaction to acoustic stimuli, as has previously been performed in previous studies [27,28]. In order to measure the tuna’s behavioral response to the different stimuli described above, three types of behavior were listed:

- B0: No response to stimuli;
- B1: Moderate response, with the tuna presenting slight changes in vertical position, swimming velocity, or swimming tilt.
- B2: Severe response referring to abrupt changes in vertical position, swimming velocity, or swimming tilt.

In order to establish a relationship between the acoustic stimuli and the behavioral variables, statistical analysis of the data was conducted. First, the assumptions necessary for the use of analysis of variance (ANOVA) were determined. In this case, although the values did not comply with the required normality for some of the variables, homoscedasticity was satisfied according to Leven’s test (*p* > 0.05). Consequently, and assuming the robustness of the method against violations in data normality (the central limit theorem states that the sample measurements must be approximately normal), the ANOVA method was the approach applied to the present analysis. To describe the three types of behavior in a quantitative manner, a linear combination of the measured parameters obtained from principal component analysis was used. That quantitative value could be used to describe B0, B1, and B2 as dependent on the measured parameters. The ANOVA test was carried out to analyze the effect of source type and source level on the tuna’s behavioral response. Levene’s test was used to test normality and homogeneity of variances in ANOVA analysis. All statistics analysis was carried out using Statgraphics Centurion XVIII^®^ [29].

## 3. Results

### 3.1. Background Tuna Behavior

The monitoring of the usual behavior of the tuna in the cage resulted in 700 h of acoustic echograms and 150 h of video recordings. As a result, it could be inferred that the tuna school usually swam in a circular or elliptical pattern [30,31] covering a large area of the cage, swimming closer to the cage nets. As expected [32], the school depth exhibited circadian rhythms. During the middle of the day, the school tended to be closer to the surface, going deeper overnight. This behavior was repeatedly observed during the period of continuous monitoring and recording. An average night/day depth difference of 2.8 m was noticed. It was also found that the school reacted to the feeding boat’s approach. Tuna were fed with frozen mackerel blocks thrown using a tube from the boat to the middle of the cage surface twice per day. As the boat approached, tuna dove downward from the surface and swam deeper even before the boat or its shadow were visible. Then, the school remained far from the surface as the boat arrived, the boat was moored beside the cage, and food was given. Only when the boat departed did the school rise up again. Figure 3 shows the echograms corresponding to the described process, where the depicted distance of the school gravity center from the cage bottom to the transducer is represented.

**Figure 8:**
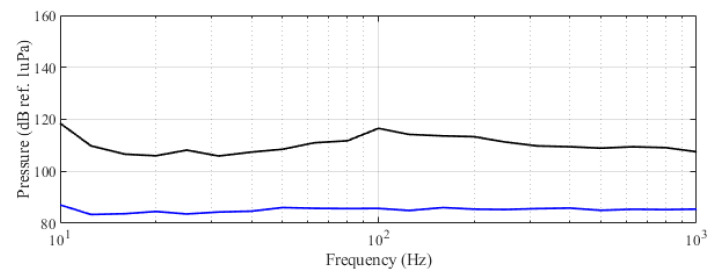
Spectra of farm acoustic landscape in third-octave levels (dB ref 1 μPa RMS). Lower curve: in the absence of operations or ships in the farm perimeter; upper curve: during maneuvers of the feeding boat upper curve.

This behavior can be interpreted in relation to feeding boat-produced noise, avoidance movement, and feeding maneuver. However, it is clear that, as expected [33], tuna reacted significantly to noise. Figure 8 shows the recorded spectra of the acoustic landscape of the farm when the feeding boat was moving alongside the cages.

### 3.2. Reaction of Tuna to Sound Playback

The farm manager authorized the experiments on the following conditions:

- Duration of the experiment is limited to a maximum of three days to avoid possible cumulative stress and prevent a decrease in tuna meat quality. In the case of a sudden rise in physiological stress indicators, some experimental time must be disposed of to ensure normal conditions before the next slaughter period.
- Maximum acoustic levels to which the tuna are commonly exposed to at the feeding installations are not exceeded.

With the aim of accomplishing the previously mentioned goals, the acoustic soundscape related to the cage was monitored. The reference conditions inferred from the conducted measurements consider the maximum sound pressure level generated by ship noise and lupara shots. In addition, a caution principle was applied regarding the duration of the signals, and it was limited to 15 s. The first assays with tone pulses were addressed to evaluate whether a panic reaction would compromise the security of the studied animals. These measurements also allowed for the determination of the presence of behavioral responses related to the threshold defined by the expected sensitive curve [16]. After a period of 48 h taking data and working with the highest SPL emissions for each single frequency, it was shown that physiological indicators in the fish slaughtered for commercial purposes did not show any significant changes that could reveal additional stress associated with the acoustical study. The signals were projected to the medium every 15 min and had a duration of 15 s. After this first trial, a second experiment was developed using longer signals with a higher averaged SPL. In the following sections, the results related to the behavioral reactions under short (from 10 to 15 s) and long (from 10 to 15 min) emission periods are summarized.

The time-table activities carried out during the observation period followed the scheme summarized below:

- The ship arrived everyday early in the morning and was moored alongside the experiment cage.
- The recording system was set up, and feeding boats approached recording tuna reactions.
- During the feeding operations, the ship’s engine was turned on to achieve maximum battery charge. The sound projector was set up.
- All measurements related to recording playbacks were developed in absence of the ship’s noise (or alternator noise) in the proximity of the cage.
- Average background measurements suggested that SPL was around 93 dB ref 1 μPa with peaks lower than 100 dB ref 1 μPa below 1 kHz. (see Figure 8 for details).
- The experiment was performed over two daily periods after tuna feeding (morning and afternoon–evening) and was repeated for 3 days.

In order to understand the results obtained in this experiment, data were analyzed per day taking into account all of the events emitted during each day. However, some of the events produced more striking reactions due to the emitted signal characteristics. For this reason, these events were studied in detail.

### 3.3. Day 1 Results

During the course of the first day of the experiment, we had the chance to test the monitoring system and the performance of the sound projector in free-field conditions and high-power emissions.

The first observations resulted from the comparison of both echosounders readings: acoustic data from the Knudsen echosounder were closer to saturation (with some values reaching it) for the given fish mean size and densities than the Biosonics echosounder data. This was a consequence of the chosen Knudsen gains, resulting in a given dynamic range for the acoustic backscattering volume strength measurements. We emitted 35 sound events that allowed us to compare the results of both echosounders when applying the described parameterization of behavior in terms of the school mass center. A discrepancy was detected between Knudsen and Biosonics recorded data, with differences in the capability of detecting slight vertical displacements of the school. The differences were primarily due to the saturated values produced by the Knudsen echosounder digital gain, which was solved on the following days. Some other differences could be attributed to the distance between two echosounders described in the measurement setup.

**Figure 9:**
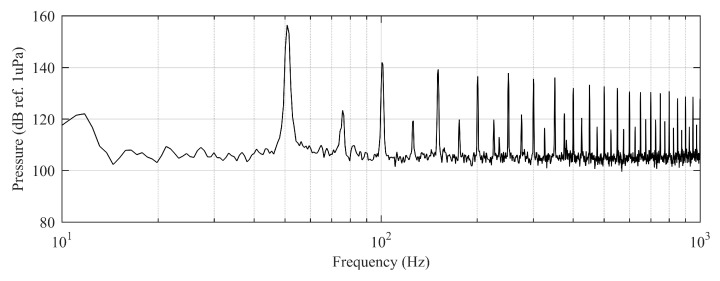
Emitted spectrum affected by loudspeaker nonlinearities for the 50 Hz pure tone excitation.

Regarding the underwater projector performance, it appeared that while reproducing pure tones at very low frequencies and the highest allowed power excitation, nonlinearities arose at the dynamic loudspeaker generating super-harmonics with enough amplitude to overlap the emitted signal (see Figure 9).

**Figure 10:**
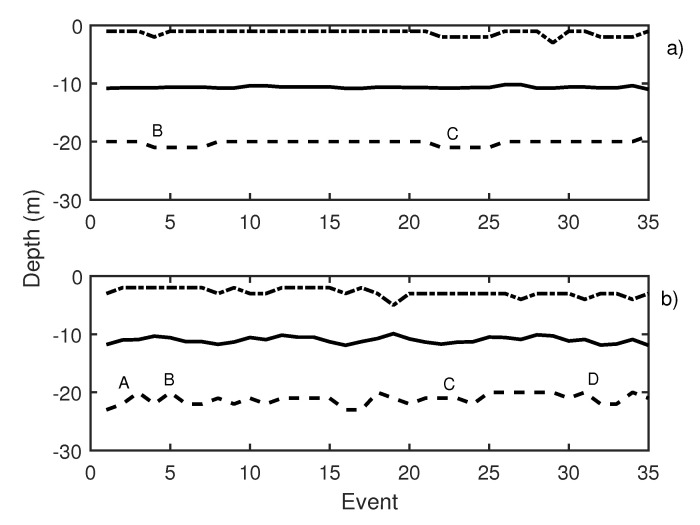
(**a**) Knudsen echosounder data recorded on day 1; (**b**) Biosonics echosounder data recorded on day 1. In both cases, the solid line represents the average depth of the school; the dashed line represents the lower limit of the school; and the point-dashed line represents the upper limit of the school.

In Figure 10, the results of the first test day are presented. This figure depicts two graphics: Knudsen echosounder data are shown in the upper part of the graphic (Figure 10a) and Biosonics echosounder in the lower part (Figure 10b). In both, the most important events are labeled. In Table 1, labeled events are described. A variation in the average depth of the school was observed in events 4 to 8 (label B in Figure 10a) that corresponds to a pure tone of 20 Hz with a increasing SPL from 140 dB (event 4) to 165 dB ref 1 μPa (event 8). The same reaction was shown in events 20 to 23 (label C in Figure 10a), in this case caused by a 500 Hz pure tone (with the same increase in the level among the events). On the other hand, for the Biosonics echosounder data (Figure 10b), the average depth and upper and lower limit variations were determined. When a 1000 Hz pure tone was emitted, the school was contracted near the surface. In Figure 10b, label A presents an increase in the lower limit of school due to the 1000 Hz pure tone. The average trace length decreased with respect to the previous situation, which could indicate an increase in the swimming velocity of the tuna. Moreover a swimming tilt change was taking place, and the tuna began to swim upwards. The 20 Hz pure tone, according to the Biosonics echosounder data, caused school expansion and contraction (label B Figure 10b). The same applied for the 500 Hz pure tone (label C Figure 10b) and windmill playback (label D in Figure 10b). This was further supported by the measurement of trace length and tilt. Trace length decreased, and, thus, swimming speed increased. In addition, a rapid change in average trace tilt occurred, changing from swimming upward to swimming downward repeatedly. This behavior is normally associated with an alarm situation that causes an increase in activity and in the expansion and closure of the school [34,35].

### 3.4. Day 2 Results

**Figure 11:**
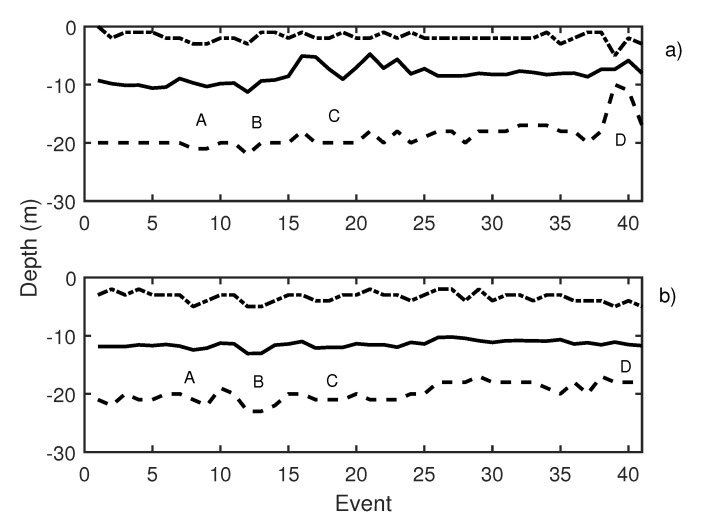
(**a**) Knudsen echosounder data recorded on day 2; (**b**) Biosonics echosounder data recorded on day 2. In both cases, the solid line represents the average depth of the school; the dashed line represents the lower limit of the school; and the point-dashed line represents the upper limit of the school.

During the second working day, 41 events were emitted. Knudsen echosounder digital gain was balanced, and different pure tones were sequentially emitted. Pure tones of 19 Hz (events 3 to 5 in Figure 11), 50 Hz (events 6 to 8 in Figure 11), and 300 Hz (events 9 to 11 in Figure 11) were reproduced using the underwater sound projector. Those pure tone emissions caused an increase in school activity that could be observed at two echosounders (Figure 11a,b, label A). In Table 2, changes in swimming tilt and length of the traces are described. Changes in the length of the traces were documented to correspond to changes in the swimming velocity of the tuna. In the same way, changes in average swimming tuna tilt were observed, and tuna swam upward and downward. These changes occurred in expansions and contractions of the school. Additional measurements were carried out by performing the acoustic emission but considering other types of noise, in this case, a lupara shot (Figure 11, label B). Specifically, the lupara emission was performed with a peak SPL of 216.4 dB (ref 1 μPa). As a result, the average depth of the tuna school and the upper and lower limits decreased. In addition, swimming tilt changed (Table 2), and, consequently, the school swam farther from the sea surface. After this moment, broadband noises were emitted. During broadband noises (label C in Figure 11) school behavior remained unstable. Contractions and expansions of the school were detected. Finally, a 50 Hz pure tone was emitted with an SPL of 165 dB ref 1 μPa. This emission was repeated from event 38 to event 40, and it is illustrated in Figure 11, label D. These events caused a large contraction of the school, so that the difference between the upper and lower limits decreased from 19 to 13 m. Results obtained on the second day indicated that tuna presented the same alarm behavior as in the previous day.

### 3.5. Day 3 Results: Windmill Recorded Playback Increases Exposure

**Figure 12:**
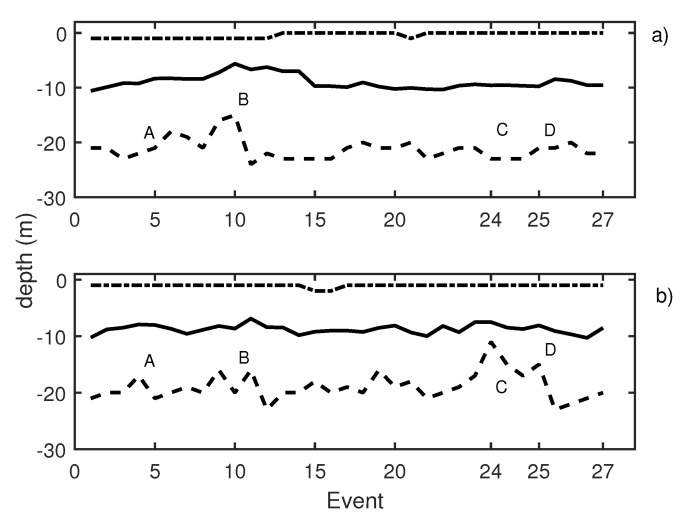
(**a**) Knudsen echosounder data recorded on day 3; (**b**) Biosonics echosounder data recorded on day 3. In both cases, the solid line represents the average depth of the school; the dashed line represents the lower limit of the school; and the point-dashed line represents the upper limit of the school.

**Figure 13:**
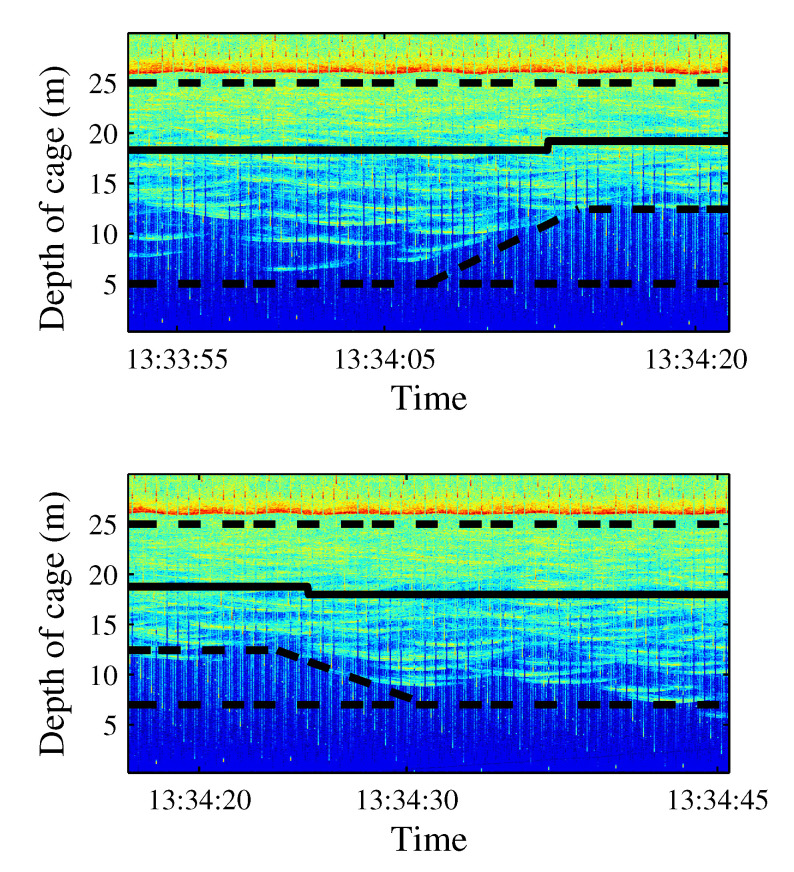
Echogram corresponding to the reaction to the first short-time (15 s) emission of a windmill recording, SPL between 110 and 145 dB. The tuna moved upward (**upper panel**) and recovered their normal distribution later (**lower panel**).

On the last measurement day, 27 events were reproduced, and the results are depicted in Figure 12. Pure tones of 30 Hz, 50 Hz, and 150 Hz were emitted in events 5 to 9 with a SPL of 185 dB ref 1 μPa (Figure 12, label A). These emissions caused school contraction and expansion. As in all other previous cases, low-frequency emissions induced higher activity of fish. The average values of trace length and trace tilt shown in Table 3 support this statement. A qualitative change occurred when reproducing wind turbine noise recordings for 15 s after a long pause in the experiments while waiting for the absence of the operational shipping noise. The results can be observed in Figure 12, label B, and detailed results of the echogram are given in Figure 13. Behavioral changes were found during the emission of turbine sound when considering the fact that the SPL equivalent was expected at a distance of 50 m from the source (windmill). The observed movement pattern of the tuna could be interpreted as a maneuver to avoid this noise. Once the sound ceased, they recovered their original distribution in the cage.

With the aim of identifying reactions to acoustic stimulus, the time of exposure to noise was increased considering turbine sound with an SPL of 182 dB being continuously emitting for 15 min (label C in Figure 12). During the first emission of sound, the tuna started to show behavioral changes after 8 min of sound exposure. The observed reaction can be described in terms of three variables, namely, the position of the school along the water column, changes in the school swimming pattern considering the size and position, and the changes in the swimming direction.

**Figure 14:**
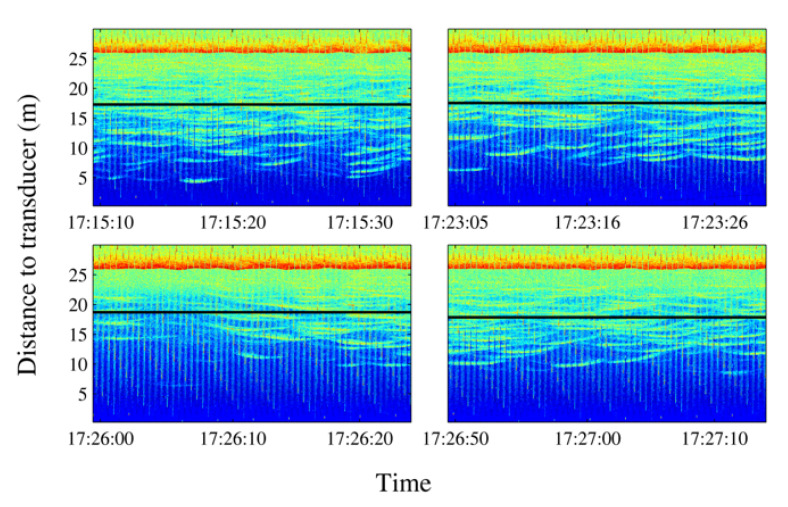
Echograms showing evolution of the average school depth during a long-time emission (10 min). The relative absence of tuna tracks in the third echogram can be noted in the left lower panel. The solid line represents average depth of the school; the dashed line represents the upper limit and the lower limit of the school. In the lower limit case, the dashed line is split to mark the real lower limit of the school versus previous lower limit.

**Figure 15:**
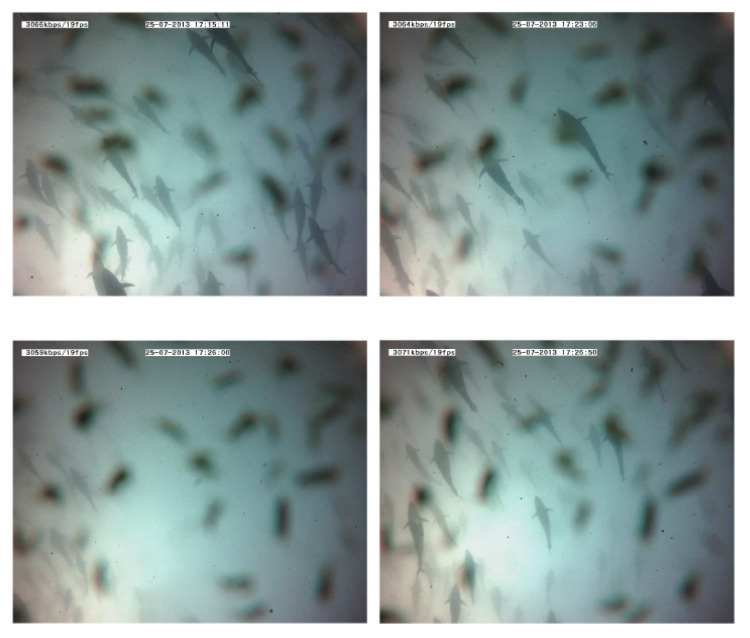
Images recorded during a 10-minute emission. The absence of tuna after a few minutes should be noted (third frame). Tuna shapes are slightly masked by the presence of copepods on the camera lens.

- School depth: a few minutes after starting the noise emission, the school moved upward (see Figure 14). The tuna remained closer to the surface even when acoustic emissions had finished, and only some minutes later did they recover their original distribution.
- Swimming pattern size and position: the tuna bunched together and swam closer to one to each other. They still acted like a school with a circular pattern but displaying circles of a smaller radius and occupying only half of the cage (see Figure 15).
- Swimming direction: to identify possible changes in swimming direction, ten random intervals of five minutes of video recordings were analyzed before and after long acoustic emissions. During the intervals before the emission, none of the tuna changed their swimming direction from that of the school. However, after a long noise emission, an average of 15 tuna individuals were registered to swim in the opposite direction at a higher speed. This reaction was observed in addition to the two changes described above.

A second acoustic emission (label D in Figure 12) was performed 30 min after the first one. In this case, the same behavioral changes were observed but the reaction appeared at minute 11 of the emission (3 min later with respect to the previous emission). A third emission was carried out assuming again 30 min to allow the tuna to recover their normal behavior. In this case, the tuna did not show any kind of reaction to the noise.

### 3.6. Statistics Analysis Results

A two-way ANOVA was conducted to examine the effect of source type and source level on behavioral variables (Table 4). There was a statistically significant interaction between the effects of source type and source level on average tilt (F(4, 94) = 4.558, *p* = 0.002) and, to a lesser extent, on average length (F(4, 94) = 2.517, *p* = 0.046). However, a simple main effects analysis only showed a significant difference in TSP and MLS sources for a high source level (SPL between 150 and 165 dB ref 1 μPa). This result may be due to the small number of samples recorded with these sources. Focusing on the independent effect of each acoustic property of the stimuli, there was a statistically significant difference in the levels for all variables (*p* < 0.05). However, a Tukey’s honest significance test (Table 5) revealed that only the average length of traces obtained for very high source level (SPL > 170 dB ref 1 μPa) was significantly lower than that for the others (47.13 ± 22.3, *p* < 0.05). This result may be due to the high levels of emitted noise causing an increase in swimming speed and, therefore, the decrease in the average length of traces. In the second case, the average tilt was clearly lower when the source was excited with a low level (SPL between 120 and 140 dB ref 1 μPa) (−4.35 ± 0.35, *p* < 0.05), which denotes a response of tuna to all of the sound stimuli used in the test with a level clearly higher than that for the background noise (Table 6). Regarding the type of source, independently, it has a significant effect on the average depth (F(6, 94) = 5.797, *p* = 0.000036) and average tilt (F(6, 94) = 14.471, *p* = 1.25 × 10${}^{-11}$). In order to establish a clear separation between the results obtained for each variable, TSP and MLS sources were removed from the analysis (number of samples = less than 2). Using a Tukey post hoc test, it was possible to establish two groups divided by sources for average depth. In this case, there was a statistically significant difference between the group of sources formed by pure tones (−9.61 ± 1.54, *p* = 0.008) and windmill noises (−9.32 ± 1.39) with regard to chirp (−5.89 ± 1.61, *p* < 0.05), as shown in Table 7. On the other hand, in Table 8, average tilt shows a clear difference between the values obtained for background noise (SPL < 120 dB ref 1 μPa) (−4.35 ± 0.35, *p* < 0.05) and other sources ($F_{tone}$: 1.35 ± 1.92; $F_{windmill}$: 3.15 ± 1.61; $F_{lupara}$: 4.80 ± 0.01; $F_{chirp}$: 4.50 ± 0.01; $F_{two-tones}$: 3.53 ± 0.67; *p* < 0.05). This result is in accordance with that obtained for the source-level analysis, that is, the lower level and smallest angle, as the background noise presents a minimum level.

On the basis of the results presented in Table 9, it can be observed that interaction between source level and source type did not cause significant differences in the behavioral reactions of the tuna. However, when the effects of source type and source level are evaluated independently, a moderate response to acoustic stimuli (B1) presents significant differences (*p* << 0.05) with both factors. Unfortunately, this does not occur for the cases of B0 and B2. The low numbers in B0 (five samples) and B2 (25 samples) measurements may explain the observed results.

## 4. Discussion

Reactions of tuna to underwater noise were identified by applying a monitoring methodology based on the combined use of vertical echosounders and video cameras, which allowed us to parameterize and quantify them. The observed reactions were related to the emission of signals with a high-power, low-frequency projector, and especially to low frequencies, pure tones, broadband noises, long exposure activities, and the highest SPL-emitted values. These reactions can be summarized as follows:

(i) Position change in the water column of the fish school.
(ii) Increase in school activity: contraction and expansion of the school (alarm).
(iii) Displacement and contraction of the school (avoidance).
(iv) After the longest emissions, some specimens swam in the opposite direction to the rest of school, which could be interpreted as slight disorientation.
(v) Increased speed.

Reactions to noise summarized above were repeatedly observed during the various emissions. Emission duration had to be increased progressively to observe similar reactions via various measurements. Therefore, for semi-captive bluefin tuna, a high degree of adaptability to noise could be considered.

To reinforce this, a statistical analysis was carried out to verify the relationship between the observed reactions and the applied stimuli. Behavioral parameters have a strong dependence on source level. On the other hand, only two parameters, average depth and average tilt angle, presented significant differences with source type. This suggests that behavioral responses are potentially affected by level regardless of the type of emitted noise. Moreover, average tilt length showed a statistically significant difference with interaction in regard to the effects of source type and source level. A deeper analysis revealed that average tilt differences were found when the sound level of stimuli was higher than that of the background noise. However, the average tilt length depends on very high levels of sound. The two-way ANOVA test results, as shown in Table 9, indicate that only moderate reactions presented significant statistical differences with source level and source type effects when they were independently evaluated. This reaction level was the most commonly found in this study, being observed in 69% of cases. Severe reactions were observed in a lower number of cases, which could be too low for a significant analysis. Emission conditions could have been forced in order to observe a higher number of severe reactions. However, this could have caused unintended damages to fishes, and this is the reason why this test was not carried out.

The current experiment proved that tuna behavior can be affected by noisy stimuli, but more exhaustive experiments should be developed to obtain a complete characterization of time and intensity thresholds and to properly determine the effect of turbine operational noise on bluefin tuna. It would be especially interesting to increase the time duration of exposure to noise as well as the SPL of the emitted signals in order to obtain results related to tuna’s adaptability to noise or accumulative stress effects. It is important to also remark that we were working with semi-captive animals, which do not have the same constraints as those of tuna in the wild (e.g., they do not need to hunt for prey; they swim in a limited space; they may have developed a level of tolerance to ship noise).

It is difficult to study the effect of human activities on the marine environment. Sometimes, the obtained results are tentative or inconclusive, but the existing lack of knowledge should encourage the development of specific studies. In relation to underwater noise, knowledge about physiology or behavioral changes is required to develop methodologies and tools that allow for the relationship between pollutants and the effect that they have to be determined. The proposed methodology offers a way to develop objective observations with acoustical and image recordings susceptible of automatic processing to parameterize behavioral changes. While the use of echosounders and video cameras (single or stereoscopic) can allow one to properly describe the produced reactions, a discussion regarding the availability of low-frequency underwater projectors for scientific studies is still necessary. In our work, and thanks to the collaboration of the Spanish Army, we had the rare opportunity to use a high-power, low-frequency projector, usually restricted to military uses (mainly for budget reasons), which offered the possibility of emitting signals with a source level above 150 dB between 20 Hz and 20 KHz. There is a general concern about the lack of proper low-frequency sources for research purposes. Piezoelectric technologies usually cannot achieve such lower frequencies, and they do not have flat frequency responses. Moreover, underwater electrodynamic loudspeakers are usually found with limitations in operational depth and have efficient frequencies only above 100 Hz and below 20 kHz. Therefore, there is a need for new commercial developments that facilitate access to military technology for marine research and assessment, including hydrostatic compensation mechanisms and high-power emissions with limited nonlinearities due to source performance. High source levels are associated with nonlinear propagation because of the high-pressure wave interacting with the medium, but undesired nonlinear oscillations due to device aging (as attributed in our case by the army maintenance service) or the oscillator’s characteristics can arise at higher power levels, altering the desired spectral content of the emitted signal. This aspect must be examined, and it can also be compensated for by characterizing the transfer function of the emitting device and properly designing the excitation signals.

In migratory species, as in the case of bluefin tuna, behavioral changes can be highly relevant to their spawning periods and routes. Considered recently a species with endangered populations, it is very important to study the habits of bluefin tuna and their possible conditioning. Bluefin tuna are extremely difficult to manipulate in captivity, and it is challenging to study them in the wild because of their great mobility. The restrictions in catching quotas also limit the access to individuals for experimentation. The activities presented in this work support the initial insight into the problems concerning the effects of anthropogenic noise and particularly offshore wind turbine noise on bluefin tuna behavior.

## Figures and Tables

**Table 1:** Description of labeled events presented in Figure 10.

Label	Event	Emitted Signal	Average Trace Length	Swimming Tilt (*)
A	1 to 3	1000 Hz	20 pings	up
B	4 to 8	20 Hz	15 pings	up/down
C	20 to 23	500 Hz	15 pings	up/down
D	30 to 34	Windmill	15 pings	up/down

(*) Swimming tilt must be understood as change in swimming tilt with respect to the previous situation.

**Table 2:** Description of labeled events presented in Figure 11.

Label	Event	Emitted Signal	Average Trace Length	Swimming Tilt (*)
A	3 to 11	19 Hz, 50 Hz, and 300 Hz	14 pings	down/up/down
B	12 to 13	lupara	11 pings	down/up
C	14 to 25	broadband noises	15 pings	down/up
D	38 to 40	50 Hz	10 pings	up/down

(*) Swimming tilt must be understood as change in swimming tilt respect to the previous situation.

**Table 3:** Description of labeled events presented in Figure 12.

Label	Event	Emitted Signal	Average Trace Length	Swimming Tilt (*)
A	5 to 9	30 Hz, 50 Hz, and 150 Hz	14 pings	up/down/up/down
B	11	windmill 15″	10 pings	down/up/down
C	24	windmill 15′	7 pings	up/down
D	25	windmill 15′	8 pings	up/down

(*) Swimming tilt must be understood as a change in swimming tilt with respect to the previous situation.

**Table 4:** Two-way ANOVA conducted on the effect of source type and source level on the behavioral variables.

Source	MS	df	AF	*p* (*)
**Upper limit**				
Source type	4.667	6	1.182	0.323
Source level	22.340	3	11.318	0.000
Source type × source level	0.375	4	0.142	0.966
Residuals	61.847	94		
**Lower limit**				
Source type	41.158	6	1.507	0.184
Source level	41.214	3	3.017	0.034
Source type × source level	14.326	4	0.951	0.438
Residuals	427.981	94		
**Average depth**				
Source type	55.048	6	5.797	0.000
Source level	27.899	3	5.876	0.001
Source type × source level	5.171	4	0.817	0.518
Residuals	15.5756	94		
**Average length of traces**				
Source type	1551.111	6	0.977	0.445
Source level	24,558.073	3	30.939	0.000
Source type × source level	2663.504	4	2.517	0.046
Residuals	24,870.886	94		
**Average tilt of traces**				
Source type	186.391	6	14.471	0.000
Source level	17.505	3	2.718	0.049
Source type × source level	39.136	4	4.558	0.002
Residuals	201.78	94		

(*) *p*-values << 0.05 = factors with a statistically significant effect with a 95.0% confidence level.

**Table 5:** Tukey post hoc test for average length. Homogeneous groups for source level.

Source Level	N	Subset 1 (**)	Subset 2 (**)
Very high (SPL > 170 dB ref 1 μPa)	30	47.13	
High (SPL = 150–165 dB ref 1 μPa)	53		93.04
Medium (SPL = 140–150 dB ref 1 μPa)	15		79.40
Low (SPL 120–140 dB ref 1 μPa)	9		75.56
Background (SPL < 120 dB ref 1 μPa)	2		77.00
Sig.		1.000	0.312

(**) Subset for alpha = 0.05.

**Table 6:** Tukey post hoc test for average tilt. Homogeneous groups for source level.

Source Level	N	Subset 1 (**)	Subset 2 (**)
Very high (SPL > 170 dB ref 1 μPa)	30		1.0367
High (SPL = 150–165 dB ref 1 μPa)	53		2.7811
Medium (SPL = 140–150 dB ref 1 μPa)	15		0.9053
Low (SPL = 120–140 dB ref 1 μPa)	9		0.8789
Background (SPL < 120 dB ref 1 μPa)	2	−4.3500	
Sig.		1.000	0.428

(**) Subset for alpha = 0.05.

**Table 7:** Tukey post hoc test for average depth. Homogeneous groups for source type.

Source Type	N	Subset 1 (**)	Subset 2 (**)
Tone	72	−9.6147	
Windmill	23	−9.3226	
Lupara	2	−9.2800	
Background	2	−8.1850	−8.1850
Two-tone	6	−8.1017	−8.1017
Chirp	2		−5.8950
Sig.		0.747	0.313

(**) Subset for alpha = 0.05.

**Table 8:** Tukey post hoc test for average tilt. Homogeneous groups for source type.

Source Type	N	Subset 1 (**)	Subset 2 (**)
Tone	72		1.3582
Windmill	23		3.1565
Lupara	2		4.8000
Background	2	−4.3500	
Two-tone	6		3.5333
Chirp	2		4.5000
Sig.		1.000	0.123

(**) Subset for alpha = 0.05.

**Table 9:** Two-way ANOVA conducted on the effect of source type and source level on the reaction responses of tuna.

Source	MS	df	AF	*p* (*)
**BO**				
Source type	1.155	1	0.07	0.835
Source level	1.108	1	0.07	0.838
Source type × Source level	0	0	0	–
Residuals	16.368	1		
**B1**				
Source type	261.8	14	2.71	0.006
Source level	867.64	3	41.98	0.800
Source type × Source level	171.7	18	1.38	0.189
Residuals	289.33	42		
**B2**				
Source type	211.401	10	1.57	0.235
Source level	89.385	1	1.63	0.125
Source type × Source level	24.93	2	0.92	0.4253
Residuals	148.239	11		

(*) *p*-values << 0.05 = factors with a statistically significant effect with a 95.0% confidence level.
